# Single-Cell RNA Transcriptomics Reveals the State of Hepatic Lymphatic Endothelial Cells in Hepatitis B Virus-Related Acute-on-Chronic Liver Failure

**DOI:** 10.3390/jcm11102910

**Published:** 2022-05-20

**Authors:** Pengpeng Zhang, Hao Li, Chen Zhou, Kai Liu, Bo Peng, Xingguo She, Ke Cheng, Hong Liu, Yingzi Ming

**Affiliations:** 1 The Transplantation Center of the Third Xiangya Hospital, Central South University, Changsha 410013, China; zpp_china@163.com (P.Z.); lihao199595@163.com (H.L.); 15073139770@163.com (C.Z.); kailiucsu@163.com (K.L.); pengbo_2000@163.com (B.P.); shexingguo@163.com (X.S.); chke1972@163.com (K.C.); hong804@sohu.com (H.L.); 2 Engineering & Technology Research Center for Transplantation Medicine of National Ministry of Health, Changsha 410013, China

**Keywords:** cirrhosis, acute-on-chronic liver failure, lymphangiogenesis, lymphatic endothelial cells, ScRNA-seq, secreted phosphoprotein 1

## Abstract

Acute-on-chronic liver failure (ACLF) is an acutely decompensated cirrhosis syndrome with high short-term mortality. Very little is known about the relationship between the lymphatic system and ACLF. We explored the role of hepatic lymphatic vessels (LVs) and lymphatic endothelial cells (LyECs) in ACLF using human liver samples with the help of single-cell RNA-sequencing (scRNA-seq) technology. Here, ACLF exhibited more severe liver injury and inflammation than cirrhosis, as indicated by significant increases in plasma levels of alanine/aspartate aminotransferases and total bilirubin. Compared with cirrhosis cases, the number of intrahepatic LVs was decreased significantly in ACLF patients. ScRNA-seq revealed that many monocyte/macrophages infiltrated into the liver of ACLF cases. Meanwhile, scRNA-seq revealed a group of apoptotic and dysfunctional LyECs, which were the result of secreted phosphoprotein 1 (SPP1) released from infiltrating monocyte/macrophages. In vitro, SPP1 increased the proportion of dead LyECs significantly and impaired the ability of tube formation of LyECs in a dose- and time-dependent manner. In conclusion, ACLF is associated with less LV and LyEC dysfunction, at least in part mediated by SPP1 released from infiltrating monocyte/macrophages. Hepatic LVs and LyECs can be a novel therapeutic strategy for ACLF.

## 1. Introduction

Acute-on-chronic liver failure (ACLF) is an acute syndrome and defined as an exacerbation of chronic liver disease (CLD), such as cirrhosis, initiated by a precipitating event (e.g., hepatitis or alcohol abuse) [1]. ACLF is characterized by multiple organ dysfunction/failure, finally resulting in a high prevalence of mortality in the short-term [2,3,4]. In Western countries, 60% of ACLF cases are closely related to alcoholism-induced cirrhosis. In Asians, ACLF is more common in patients with hepatitis B-related cirrhosis [5]. Chronic hepatitis B virus (HBV)-related ACLF accounts for >80% of all cases in China, which places a tremendous burden on the health care system [6]. Allogeneic liver transplantation is the most efficacious treatment for ACLF patients. However, the expense of allogeneic liver transplantation and limited liver sources prevents many suitable patients from receiving timely treatment [7]. New and efficacious medical methods are needed urgently to attenuate the incidence and mortality of ACLF.

As the most important lymph-producing organ, the liver accounts for 50% of the body’s lymph fluid [8,9,10]. In recent years, research on lymphatic vessels (LVs) and lymphangiogenesis has progressed rapidly because of the discovery of specific markers for LyECs. However, the role of lymphatic system in liver diseases has not been explored deeply [10]. LVs maintain tissue fluid homeostasis by working with the vascular system through the transport and reabsorption of interstitial fluid and lipids. However, LVs also help with the “trafficking” of immune cells into lymph nodes to accelerate inflammation resolution [8,10,11,12,13]. In patients suffering from liver cirrhosis, lymph production increases up to 30-fold and is accompanied by lymphangiogenesis, which shows the importance of LVs in hepatic inflammatory diseases [10]. Scholars have reported that impaired lymphatic drainage in rats suffering from liver cirrhosis and treatment with a nitric oxide synthase inhibitor can improve lymphatic drainage and reduce ascites volume [14]. Observations in humans and rats reveal the importance of LVs in CLD. Even though LVs have been shown to play an important role in liver diseases, their state and function in ACLF are not known. Given the role of inflammation in ACLF, it is tempting to speculate that controlling LVs may help to reduce inflammation in chronic liver cirrhosis and prevent its development into ACLF.

The innate immune system plays a major contributory role in ACLF development. Activated Kupffer cells (KCs) secrete pro-inflammatory cytokines (e.g., tumor necrosis factor (TNF)-α, interleukin (IL)-1β, and IL-6), chemokines, and reactive oxygen species (ROS) that amplify the pro-inflammatory signal and increase infiltration of immune cells (mainly macrophages and neutrophils) into the liver, further contributing to the inflammatory process [15,16]. Among the various immune cells, macrophages interact most with LVs [10]. LyECs secrete chemotactic factors to attract macrophages [17]. In turn, macrophages secrete lymphangiogenic cytokines such as vascular endothelial growth factor (VEGF)-C, VEGF-D, and VEGF-A to promote inflammation-induced lymphangiogenesis [18]. It has been postulated that patients with ACLF display evidence of a pro-inflammatory state, with local hepatic inflammation and vascular endothelial dysfunction that accelerates progression to multiple organ failure [19]. Therefore, new therapeutic approaches to reduce intrahepatic infiltration of macrophages may reduce the incidence and mortality of ACLF. In-depth exploration of the relationship between intrahepatic LVs and infiltrating macrophages could provide a solid theoretical foundation.

Single-cell RNA sequencing (scRNA-seq) technology permits comparison of the transcriptome at the single-cell level as well as evaluation of the transcriptional differences and similarities in cell populations [20]. With its extremely high resolution, scRNA-seq can distinguish cell heterogeneity, as well as exploring and defining rare cell subgroups, especially in the exploration of immune-related diseases [21]. For example, Iwakiri et al. revealed zone-specific alterations of hepatic sinusoidal endothelial cells in liver cirrhosis by scRNA-seq [22]. At the same time, scRNA-seq can also be used to restore the developmental lineage between cells and predict key regulatory transcription factors during differentiation [21]. This ability enables understanding of the complex regulatory network in the pathological process and revelation of the key pathogenic mechanisms in disease, aiding the discovery of potential therapeutic targets.

Here, we used scRNA-seq to explore changes in the state and function of LyECs between HBV-related cirrhosis and ACLF. Comparison of the LVs and state of LyECs between cirrhosis patients and ACLF revealed that ACLF patients had fewer LVs, accompanied by apoptotic and dysfunctional LyECs. We hypothesized that LVs and state of intrahepatic LyECs might be an important reason for the development of cirrhosis into ACLF. We intended to explore the pathophysiological mechanism of ACLF and find novel interventions to reduce the incidence and mortality of ACLF.

## 2. Materials and Methods

### 2.1. Study Population and Collection of Human Liver Samples

The present study involved 25 human liver samples: 5 from healthy controls (HCs), 10 from cirrhosis patients, and 10 from ACLF patients. The liver samples from HCs were derived from cardiac death donors. The cirrhosis patients and ACLF patients were HBV-related. The diagnostic criteria of all ACLF patients met the definitions developed by the European Association for the Study of the Liver–Chronic Liver Failure Consortium (EASL-CLIF) [4] and the definition developed by the Chinese Group on the Study of Severe Hepatitis B (COSSH) [23]. The exclusion criteria were as follows: 1. Hepatitis B virus (HCV) infection or complication with HCV infection; 2. Individuals with hepatocellular carcinoma. 

### 2.2. Liver Tissue Dissociation and Preparation

Fresh liver tissues were stored in GEXSCOPE^®^ Tissue Preservation Solution (Singleron Biotechnologies, Nanjing, China) and transported to the Singleron lab on ice as soon as possible. Specimens were washed thrice with Hank’s Balanced Salt Solution (HBSS) and minced into pieces of size 1–2 mm. Then, the tissue pieces were digested with 2 mL GEXSCOPE^®^ Tissue Dissociation Solution (Singleron Biotechnologies, Nanjing, China) for 15 min at 37 °C in a 15 mL centrifuge tube with sustained agitation. After digestion, 70 um sterile strainers were employed to filter samples. Next, we centrifuged samples at 50× *g* for 5 min at 4 °C to remove hepatocytes [24,25,26,27]. Then, the precipitation was discarded, and the supernatant was resuspended in 1 mL of phosphate-buffered saline (PBS; HyClone, Logan, UT, USA). This action was followed by centrifuging the samples at 300× *g* for 5 min at 4 °C. Then, the supernatant was discarded, and the precipitation was resuspended in 1 mL of PBS. To remove red blood cells, 2 mL GEXSCOPE^®^ red blood cell lysis buffer (Singleron Biotechnologies, Nanjing, China) was added for 10 min at 25 °C. The solution was then centrifuged at 500× *g* for 5 min at 4 °C and resuspended in PBS. The samples were stained with trypan blue (Sigma, Darmstadt, Germany) and evaluated under a microscope. These cells were hepatic NPCs with few hepatocytes.

### 2.3. scRNA-seq and Primary Analyses of Raw Read Data

Single-cell suspensions of 1 × 10^5^ NPC cells/mL concentration in PBS were prepared and then loaded onto microfluidic devices. ScRNA-seq libraries were constructed using the GEXSCOPE^®^ Single-Cell RNA Library Kit (Singleron Biotechnologies, Nanjing, China) [28]. Individual libraries were diluted to 4 nM and pooled for sequencing. Pools were sequenced on Illumina HiSeq X with 150 bp paired-end reads. Raw reads were processed with fastQC and fastp to remove low-quality reads. Poly-A tails and adaptor sequences were removed by cutadapt. After quality control, reads were mapped to the reference genome GRCh38 (ensembl version 92 annotation) using STAR. Gene counts and unique molecular identifier (UMI) counts were acquired via featureCounts software. Expression matrix files for subsequent analyses were generated based on gene counts and UMI counts.

### 2.4. Quality Control, Dimension Reduction, and Clustering

Cells were filtered by gene counts >200 and UMI counts <6000. Cells with >25% mitochondrial content were removed. After filtering, 26,200 cells and 24,194 genes were retained for downstream analyses. We used functions from Seurat v4.0 for dimension reduction and clustering [29].

### 2.5. Analyses of Signaling-Pathway Enrichment Using the Kyoto Encyclopedia of Genes and Genomes (KEGG) Database

The potential functions of endothelial/epithelial clusters were investigated using KEGG analyses, which were used with the “enrichplot” R package version. 

### 2.6. Ligand/Receptor Analyses

We wished to investigate the potential interaction between endothelial/epithelial clusters and monocyte/macrophage clusters. Ligand/receptor analyses were conducted with the “iTalk” and “CellChat” R package version. In conclusion, high-expression genes (top 50%) were included in this analyses. The origin of the key gene was shown by CellChat.

### 2.7. Measurement of Plasma Alanine Aminotransferase (ALT), Aspartate Aminotransferase (AST), Total Bilirubin (TBil), and International Normalized Ratio (INR)

To assess the severity of hepatocyte injury, we measured the plasma levels of ALT, AST, and TBil using a HITACHI 7600 Automatic Analyzer (Tokyo, Japan, U/L). We detected coagulation function by using PUN-2048A Automatic Analyzer (Beijing, China).

### 2.8. Immunohistochemical (IHC) Staining

Liver samples were fixed in 10% neutral buffered formaldehyde for 48 h at 4 °C. Paraffin blocks were created and 4 μm slides were deparaffinized with xylene and rehydrated by washing through a graded series of alcohol solutions to deionized water. The hydrated tissue sections were washed with PBS. To retrieve antigens, the sections were incubated with 10 mM of EDTA buffer (pH 6.0) for 15 min at approximately 100 °C. Sections were incubated overnight at 4 °C with primary antibody D2/40 (MAB-05667, MXB, Fuzhou, China), and excess primary antibody was washed away with PBS. Non-specific antigens were blocked by catalase for 20 min, followed by incubation with secondary antibody for 30 min. The target antigen was developed by DAB horseradish peroxidase color development methods (Kit-0014, MXB, Fuzhou, China).

### 2.9. Isolation of Intrahepatic Monocyte/Macrophages

Isolation of hepatic monocyte/macrophages was carried out as described previously [24,30]. In brief, after liver samples had been dissociated into single-cell suspensions, we used 70 um sterile strainers to filter the samples and centrifuged the samples at 50× *g* for 5 min to remove hepatocytes at 4 °C. Then, we centrifuged the supernatant at 640× *g* for 5 min at 4 °C. After discarding the supernatant, we resuspended the pellet in 4 mL of RPMI 1640 medium containing 10% fetal bovine serum. We used CD14 MicroBeads (130-050-201, Miltenyi Biotec, Cologne, Germany) to purify the monocyte/macrophages and plated them in a 12-well plate. Finally, the 12-well plate was placed in an incubator overnight to enable monocyte/macrophage adhesion.

### 2.10. Cell Immunofluorescence Staining

Hepatic monocyte/macrophages adhered to slides and were fixed by paraformaldehyde, followed by rinsing with PBS. The fixed monocyte/macrophages were permeabilized with 0.1% Triton X100 for 10 min and blocked with 5% donkey serum (100-151, Sacramento, CA, USA) for 1 h. Next, they were incubated with primary antibodies (CD14 MA1-23611, Invitrogen, Carlsbad, CA, USA), secreted phosphoprotein 1(SPP1) (ab8448, Abcam, Cambridge, UK), and CD68 (ab125212, Abcam, Cambridge, UK) overnight at 4 °C. After that, monocyte/macrophages were treated with secondary antibody (donkey anti-rabbit Alexa488, 1:300 dilution; donkey anti-mouse Alexa 568, 1:300 dilution; Invitrogen) for 30 min. Then, 4′,6-diamidino-2-phenylindole (DAPI;71010100, Biosharp, Hefei, China) was used to label nuclei. An anti-fluorescence attenuating agent (S2100, Solarbio, Beijing, China) was employed to mount the slides. 

### 2.11. H&E Staining

Paraffin sections were deparaffinized with xylenes and rehydrated by washing through a graded series of alcohol solutions to deionized water. Sections were stained by hematoxylin for 5 min and eosin for 10 min, and washed with warm water for 5 min. Then, sections were dehydrated by washing through a graded series of alcohol solutions to xylene and mounted with neutral resin (20200206, YiYang, Shanghai, China). 

### 2.12. Masson Staining

Paraffin sections were deparaffinized with xylenes and rehydrated by washing through a graded series of alcohol solutions to deionized water. Sections were stained by hematoxylin for 5 min to label nuclei and washed with deionized water. Then, sections were stained using a Masson Tricolor Staining Kit (MST-8003, MXB, Fuzhou, China) according to the manufacturer’s instructions. 

### 2.13. Acquisition and Quantification of Images

Images were documented using a DMI 3000B system (Leica Microsystems, Wetzlar, Germany). For quantification of SPP1, D2-40, and CD14, ≥10 images (×100 magnification) were obtained per slide. The quantification of SPP1 expression, the number of LVs, and CD14-positive cells were measured by Image J (National Institutes of Health, Bethesda, MD, USA). The number of LVs was subjected to statistical analyses. Lymphangiogenesis was defined as the increased numbers of LVs/portal vein (PV) area.

### 2.14. Quantitative Real-Time Polymerase Chain Reaction (qRT-PCR)

Total RNA was extracted from liver tissues using a Total RNA Extraction kit (BSC52S1B, BioFlux, Beijing, China). Extracted total RNA (1000 ng) was used as a template for reverse transcription into cDNA using a Reverse Transcript Reagents Kit (11123ES60, YEASEN, Shanghai, China). Synthesized cDNA was mixed with iTaq Universal SYBR green master mix (11201ES08, YEASEN, Shanghai, China) and amplified by a Real Time ABI 7500 PCR system (Applied Biosystems, Foster City, CA, USA) with the primers listed in Table S1.

### 2.15. Culture of Human LyECs and SPP1 Treatment

Human LyECs were purchased from Synthbio (HSC-00020121, Synthbio, Hefei, China). LyECs were cultured with Endothelial Cell Growth Medium (HSC-0030, Synthbio, Hefei, China) containing 5% fetal bovine serum (FBS) at 37 °C in an atmosphere of 5% CO_2_ in a humidified incubator. A suspension of LyECs was loaded onto a 12-well plate (1 × 10^5^ cells/well) and SPP1 (HSC-0812, Synthbio, Hefei, China) was added to medium at 200 ng/mL or 1000 ng/mL for 24 h.

### 2.16. Flow Cytometry

After LyECs were trypsinized, they were centrifuged at 1000 rpm for 5 min at room temperature. Then, the supernatants were discarded, and the pellets were resuspended in 100 μL of PBS. We added 5 uL 7-AAD (00-6993-50, Invitrogen, CA, USA) for staining for 10 min at room temperature and detected the number of 7-AAD-positive cells by flow cytometry.

### 2.17. Tube Formation Assay for LyECs

The ability of LyECs to form capillary-like structures was analyzed as described previously [31,32,33]. In brief, Matrigel^®^ growth factor-reduced basement membrane matrix (10 mg/mL, 354230, Corning, NY, USA) was loaded onto a 24-well plate by 289 μL each. The latter was allowed to incubate for 30 min at 37 °C. The suspension of LyECs with Endothelial Cell Growth Medium was prepared at 4 × 10^5^ cells/mL. Then, 250 µL of the LyECs suspension was mixed with 250 µL of control medium or SPP1 mixed medium, and loaded on Matrigel (1 × 10^5^ cells/well). Treated LyECs were cultured in a humidified incubator at 37 °C. Cell images (×100 magnification) were obtained for each well using the DMI 3000B system (Leica Microsystems, Wetzlar, Germany). The quantification of tube formation assay was subjected to total length of tube, which was analyzed by Image J (National Institutes of Health, Bethesda, MD, USA) using Angiogenesis Analyzer (http://image.bio.methods.free.fr/ImageJ/?Angiogenesis-Analyzer-for-ImageJ&lang=en, accessed on 17 March 2020).

### 2.18. Statistical Analysis 

Data are expressed as mean ± standard error of mean (SEM). Comparison between two groups was carried out using the unpaired Student’s *t*-test. Comparison between multiple groups was undertaken using one-way ANOVA. *p* < 0.05 was considered significant.

### 2.19. Ethics Statement

The study protocol was approved (2020-S023) by the Ethics Committee of the Third Xiangya Hospital of Central South University (Changsha, China). Written informed consent was obtained from all study participants. Experiments were carried out in accordance with the ethical guidelines set by the Declaration of Helsinki 1964 and its later amendments.

## 3. Results

### 3.1. ACLF Patients Exhibited More Severe Liver Damage and Inflammation and Fewer LVs than Cirrhosis Patients

**Figure 1:**
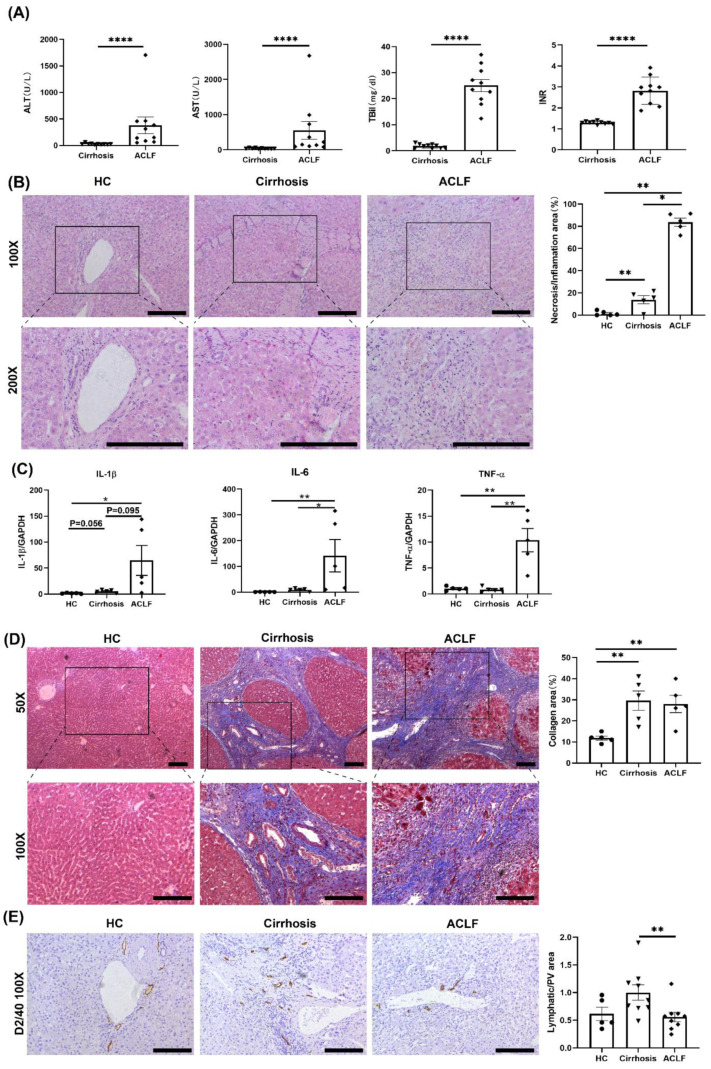
**ACLF patients exhibited more severe liver damage and inflammation and fewer LVs than cirrhosis patients.** (**A**) The plasma level of ALT, AST, TBil, and INR of enrolled 25 patients. (**B**) H&E staining of liver tissue among HCs, cirrhosis patients, and ACLF patients. (**C**) The mRNA expression of proinflammatory cytokines (IL-1β, IL-6, and TNF-α) in the liver of ACLF compared to that in cirrhosis and HC group by qRT-PCR. (**D**) Masson staining of liver tissues among HCs, cirrhosis patients, and ACLF patients. (**E**) Representative IHC images of LVs (D2/40) in the liver among HCs, cirrhosis patients, and ACLF patients (100× field) (* *p* < 0.05, ** *p* < 0.01, **** *p* < 0.0001; bar = 200 μm).

The clinical data of cirrhosis patients and ACLF patients are summarized in Table S2. We measured the plasma levels of ALT, AST, TBil, and INR. Compared with those in cirrhosis patients, the plasma levels of ALT, AST, TBil, and INR in ACLF patients were increased significantly (Figure 1A). H&E staining among HCs, cirrhosis patients, and ACLF patients revealed a larger area of hepatic parenchymal necrosis and inflammatory cells infiltration in ACLF patients than those in HCs or cirrhosis patients (Figure 1B). According to qRT-PCR, levels of proinflammatory cytokines (IL-1β, TNF-α and IL-6) were extremely high in ACLF patients, which suggested a hyperinflammatory state in the liver of ACLF patients (Figure 1C). Masson staining showed significant deposition of collagen in patients with cirrhosis and ACLF compared with that in HCs (Figure 1D), which implied that ACLF progressed from liver cirrhosis.

Considering the close association between inflammation and LVs [12], we evaluated LVs in the liver via D2/40 staining. Compared with that in cirrhosis patients, the number of intrahepatic LVs was decreased significantly in ACLF patients (Figure 1E). 

### 3.2. scRNA-seq Revealed the Infiltration of Many Monocyte/Macrophages into the Liver of ACLF Patients 

**Figure 2:**
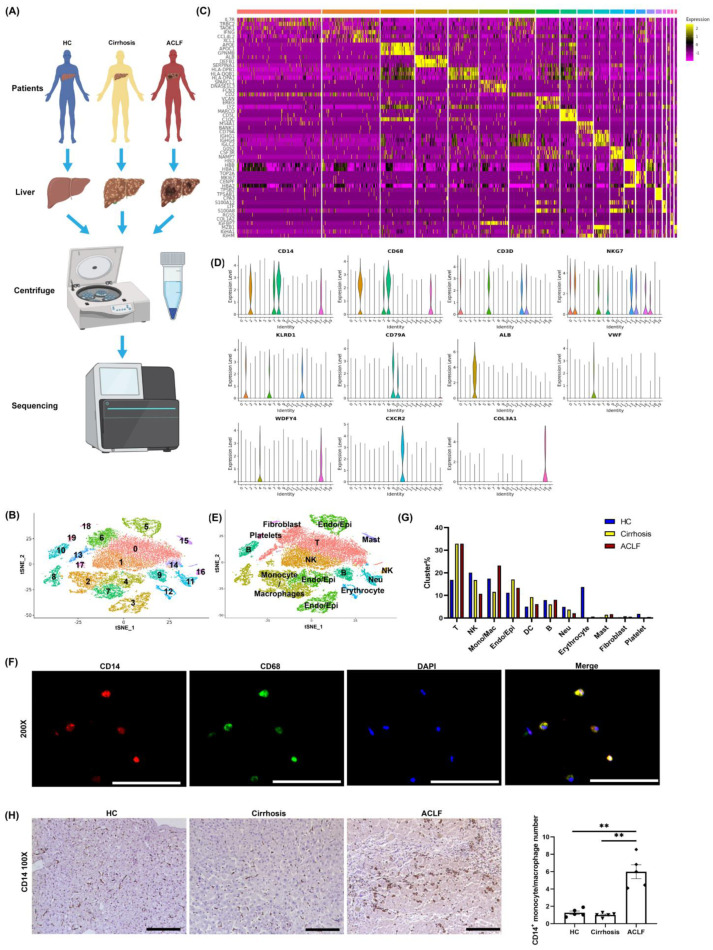
**scRNA-seq revealed that a large number of monocyte/macrophages infiltrated into ACLF liver**. (**A**) The workflow of scRNA-seq analyses. NPCs isolated from 5 ACLF livers, 3 cirrhosis livers, and 2 HC livers were included in this study. (**B**) 26,200 cells from HC (*n* = 2), cirrhosis (*n* = 3), and ACLF (*n* = 5) human livers were divided into 20 clusters (0–19), shown by tSNE plotting. (**C**) The representative marker genes of 20 subpopulations are presented with a heat map. Each column represents a different cell cluster and is arranged in the order of 0–19. (**D**) The classical marker genes of each cluster: CD14 and CD68 for monocyte/macrophages, CD3D for T cells, CD79A for B cells, NKG7 and KLRD1 for NK cells and T cells, ALB and VWF for epithelial tissue cells (including epithelial cell and endothelial cells), CXCR2 for neutrophils, WDFY4 for dendritic cells, COL3A1 for fibroblasts. (**E**) We re-annotated the 20 subpopulations according to their marker genes, and 11 classical clusters were annotated to facilitate the next analyses. (**F**) Cell immunofluorescence of CD14 and CD68 in isolated monocyte/macrophages from ACLF liver (200× field). (**G**) The proportion of 11 classical clusters among HCs, cirrhosis patients, and ACLF patients. (**H**) IHC staining for monocyte/macrophages (CD14) in the liver of HCs, cirrhosis patients, and ACLF patients (100× field). (** *p* < 0.01; bar = 200 μm).

To further analyze the intrahepatic inflammatory environment among three groups, scRNA-seq was done on non-parenchymal cells isolated from the liver tissues of the HCs, cirrhosis patients, and ACLF patients. In total, the liver samples of 5 ACLF patients, 3 cirrhosis cases, and 2 HCs were included in this study. Figure 2A illustrates the workflow of scRNA-seq. The cell viability and quality control data of scRNA-seq are shown in Figures S1 and S2, respectively. After hepatocyte sedimentation via low-speed centrifugation and removal of suspicious double cells and low-activity cells through quality control, 26,200 cells were included for further analyses. After re-clustering these 26,200 cells, we identified 20 subpopulations (0–19) that contained cells from the HC, cirrhosis, and ACLF groups, with significant inter-group differences (Figure 2B). The representative marker genes of these subpopulations are presented as a heat map (Figure 2C). To annotate these populations, we analyzed the distribution of classical markers in each population (Figure 2D). Finally, 11 classical clusters were annotated to facilitate the next analysis (Figure 2E). Among them, we defined CD14^+^CD68^+^ co-expressed cells as monocyte/macrophages (Figure 2D) and we also identified that monocyte/macrophages extracted from the liver of ACLF patients co-expressed CD14 and CD68 in vitro (Figure 2F). Compared with the cirrhosis group, the ACLF group had a significantly higher number of monocyte/macrophages (Figure 2G). Meanwhile, IHC staining of CD14 revealed the massive infiltration of monocyte/macrophages into the liver of ACLF patients (Figure 2H).

### 3.3. scRNA-seq Revealed Apoptosis and Dysfunction of Hepatic LyECs in ACLF Patients

**Figure 3:**
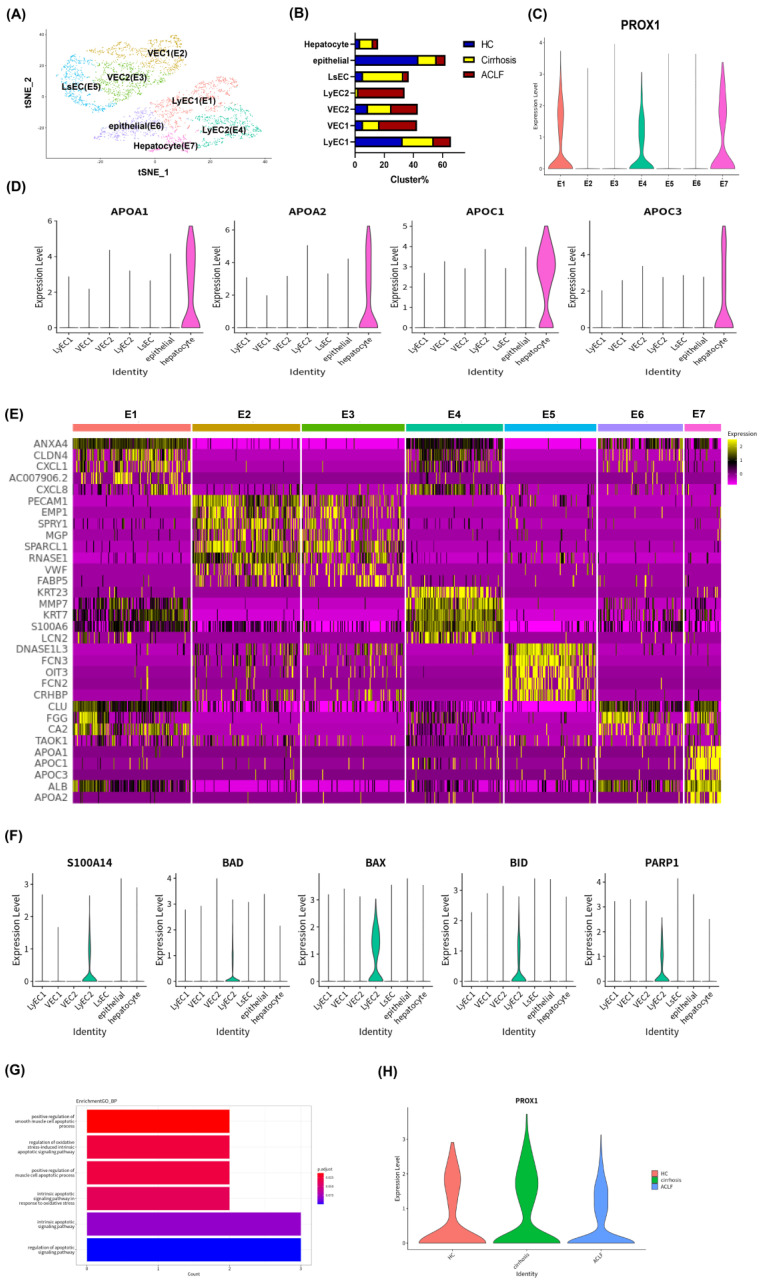
**scRNA-seq revealed apoptosis and dysfunction of LyECs in the liver of ACLF patients.** (**A**) 3769 Epithelial/endothelial cells from HCs (*n* = 2), cirrhosis patients (*n* = 3), and ACLF (*n* = 5) patients were divided into 7 clusters, which were annotated as LyEC1 (E1), vascular endothelial cell 1 (VEC1, E2), vascular endothelial cell 3 (VEC3, E3), LyEC2(E4), liver sinusoidal endothelial cell (LsEC, E5), epithelial cells (E6), and hepatocytes (E7). (**B**) The proportion of 7 clusters among HCs, cirrhosis patients, and ACLF patients. (**C**) The expression level of PROX1 in 7 clusters by violin plotting. (**D**) The expression level of hepatocyte markers (APOA1, APOA2, APOC1, and APOC3) in 7 clusters by violin plotting. (**E**) The expression of marker gene signatures for each cluster shown by heat maps. (**F**) The expression level of apoptosis-related genes in 7 clusters shown by violin plotting. (**G**) The GO biology process (BP) analyses indicated that the LyEC2 cluster in ACLF was mainly involved in apoptotic processes. (**H**) The expression level of PROX1 of LyECs among the three groups by violin plotting.

To explore the function and state of intrahepatic LyECs among the three groups, we analyzed subpopulation of endothelial cells and epithelial cells by scRNA-seq. 3769 endothelial cells, and epithelial cells were classified into E1–E7 clusters (Figure 3A). The proportion of each cluster among the three groups is shown in Figure 3B. 

PROX1 was a typical marker and functional gene for LyECs, but also expressed in hepatocytes [10,34,35,36,37,38]. In our study, E1, E4, and E7 clusters specifically expressed PROX1 (Figure 3C). To distinguish these three clusters, we enriched the highly expressed genes of each cluster by KEGG analyses to describe the functional characteristics of each cluster. As shown in Figure S3, the E7 cluster showed the characteristics of hepatocytes with the expression of a series of metabolic-related pathways, and the violin plotting showed that the E7 cluster highly expressed APOA1, APOA2, APOC1, and APOC3 (Figure 3D), which indicated that the E7 cluster was a hepatocyte population. 

Meanwhile, E1 and E4 clusters exhibited similar signaling pathways, indicating that they were clusters with similar functions, and were annotated as “LyEC1” and “LyEC2”, respectively (Figures S4 and S5). Other clusters were annotated by the classical marker shown in Figure S6. Moreover, the heat map shows that LyEC1 and LyEC2 clusters had similar genetic characteristics and profiles, but with different highly expressed genes (Figure 3E), which suggests that they were in different states. The LyEC2 cluster was present almost exclusively in the liver of ACLF patients (Figure 3B), which revealed a functional alteration of hepatic LyECs in ACLF. By KEGG analyses, the LyEC2 cluster tended to show high expression of the genes related to the oxidative phosphorylation pathway (Figure S7), which was related to stress [39,40]. The oxidative phosphorylation-related genes in LyECs of the ACLF and cirrhosis groups are shown in Figure S8. We also analyzed a series of genes related to apoptosis (S100A14, BAD, BAX, BID, and PARP1) [41,42,43], which were activated in response to acute stress and injury to cells. The LyEC2 cluster showed significantly high expression of these genes (Figure 3F). Meanwhile, the Gene Ontology (GO) biology process (BP) analyses indicated that the LyEC2 cluster in ACLF was mainly involved in apoptotic processes (Figure 3G). 

In addition, the violin plotting of PROX1 showed that the expression level of PROX1 of LyECs in the ACLF group decreased to 61% of that in the cirrhosis group (Figure 3H). All above evidence indicate that LyECs might suffer from apoptosis and dysfunction in the liver of ACLF patients.

### 3.4. Infiltrating Monocyte/Macrophages Secreted SPP1 to Induce LyECs Apoptosis and Dysfunction in the Liver of ACLF Patients

**Figure 4:**
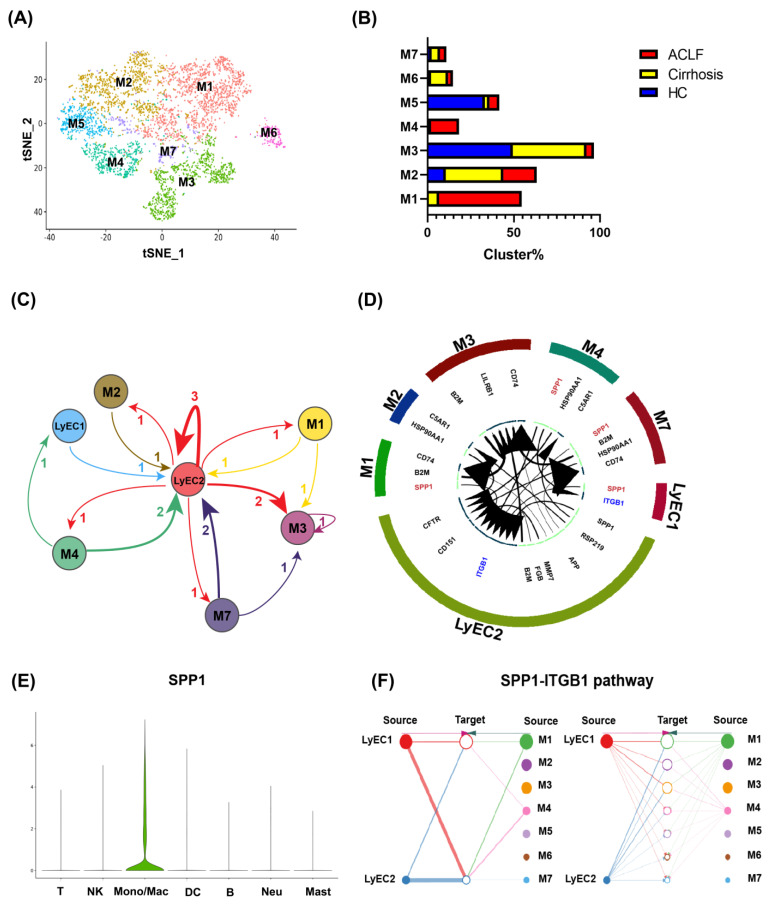
**Infiltrating monocyte/macrophages secreted SPP1 to induce apoptosis and dysfunction of LyECs in the liver of ACLF patients**. (**A**) 4738 monocyte/macrophages from HCs (*n* = 2), cirrhosis patients (*n* = 3), and ACLF patients (*n* = 5) were divided into 7 clusters (M1-M7). (**B**) The proportion of 7 monocyte/macrophage clusters. (**C**) Receptor/ligand analyses of LyECs and monocyte/macrophages. The number on the middle arrow represents the number of highly expressed (>50%) receptors and ligands that could be paired. (**D**) The key receptor/ligand for LyECs and monocyte/macrophages. The larger the area of the arrow, the higher the expression level of the receptor/ligand on this subpopulation. (**E**) SPP1 mainly originated from intrahepatic monocyte/macrophages. (**F**) M1 and M4 clusters in ACLF were the main origin of SPP1. The thicker the line, the more SPP1 secreted.

**Figure 5:**
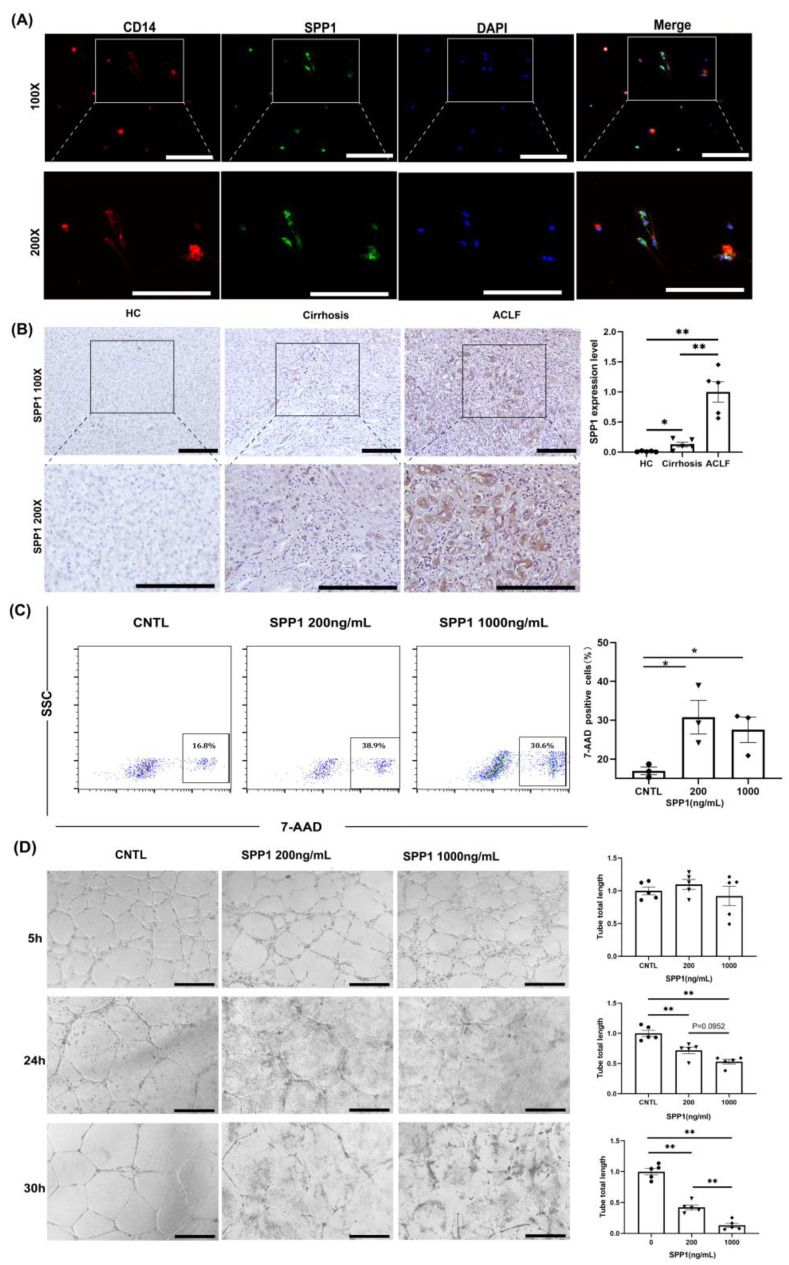
**SPP1 secreted by infiltrating monocyte/macrophages induced LyEC death and impaired the ability of tube formation of LyECs in vitro**. (**A**) Cell immunofluorescence of CD14 and SPP1 in isolated monocyte/macrophages from ACLF liver (100× and 200× field). (**B**) Representative IHC images of SPP1 in liver tissues among HCs, cirrhosis patients, and ACLF patients (100× and 200× field). (**C**) Flow cytometry showed a higher proportion of 7-AAD-positive LyECs after SPP1 treatment (200 ng/mL and 1000 ng/mL). (**D**) The tube formation assays of LyECs for CNTL and SPP1 treatment (200 ng/mL and 1000 ng/mL) (100× field). (* *p* < 0.05, ** *p* < 0.01; bar = 200 μm).

ACLF patients had more severe inflammation and monocyte/macrophage infiltration than that in HCs and cirrhosis patients. We hypothesized that infiltrating monocyte/macrophages in ACLF patients were involved in LyECs dysfunction. Further analyses of monocyte/macrophage subpopulations showed 7 different clusters (Figure 4A). The proportions of M1 and M4 clusters were increased significantly in the ACLF group (Figure 4B). Then, we analyzed the potential regulatory network between monocyte/macrophages and LyECs through ligand/receptor analyses. By matching the highly expressed ligands/receptors between monocyte/macrophages and LyECs, we found that the LyEC2 cluster was the core of the interaction network between monocyte/macrophages and LyECs (Figure 4C). Further analysis showed that monocyte/macrophages regulated LyEC2 clusters mainly through the SPP1/ITGB1 pathway (Figure 4D) and that SPP1 was secreted mainly by monocyte/macrophages in ACLF liver (Figure 4E). We also explored the source of SPP1 in monocyte/macrophage subpopulations and found that M1 and M4 clusters were the main origin (Figure 4F and Figure S9). Cell immunofluorescence also identified that monocyte/macrophages extracted from the liver of ACLF patients expressed SPP1 (Figure 5A). qRT-PCR and IHC staining of SPP1 showed that SPP1 expression in ACLF liver was extremely high compared with that in HC and cirrhosis livers (Figure S10 and Figure 5B). 

We wished to ascertain if SPP1 directly caused death or dysfunction to LyECs. We treated LyECs with SPP1 (200 or 1000 ng/mL) in vitro and counted the number of dead LyECs. Treated LyECs were stained with 7-AAD and detected by flow cytometry. SPP1 increased the death rate of LyECs significantly, which indicated that SPP1 could damage LyECs directly (Figure 5C). We also carried out tube formation assays to assess LyECs’ function in vitro. SPP1 treatment impaired the ability of tube formation of LyECs in a dose- and time-dependent manner (Figure 5D).

## 4. Discussion

ACLF is an acutely decompensated cirrhosis syndrome with high short-term mortality in 50%–90% of cases [23,44]. The pathophysiology of ACLF is still largely unknown. The importance of our study was in identification of a systemic hyperinflammatory state with monocyte/macrophage infiltration, fewer LVs, apoptosis and dysfunction of LyECs, and demonstration of the relationship between monocyte/macrophages and the lymphatic system in ACLF livers. We found that ACLF exhibited more severe damage and inflammation to the liver than cirrhosis. Meanwhile, intrahepatic lymphangiogenesis was decreased in ACLF patients than that in cirrhosis patients, and the state of LyECs in the ACLF group was apoptotic and dysfunctional because of SPP1 secreted by infiltrating monocyte/macrophages. To our knowledge, this is the first time that the characteristics and function of LVs and LyECs in ACLF patients have been demonstrated. Our findings would be useful for the development of efficacious and novel therapeutic strategies targeting LVs and LyECs in ACLF.

Recent advances in our understanding of the pathophysiological basis of ACLF indicated that a systemic hyperinflammatory state was the main driver of liver and other organ injury in patients, with cirrhosis developing into ACLF [45,46]. The greater the intensity of systemic inflammation, the larger the number of organ failures at enrollment and the higher the short-term mortality [45]. Excessive inflammation induced by Pathogen-Associated Molecular Patterns (PAMPs) and Damage-Associated Molecular Patterns (DAMPs) are considered to be important causes of tissue damage during ACLF [47]. Additionally, those inflammatory cells in the liver continuously respond to PAMPs and DAMPs, which is a feedback mechanism that promotes ACLF [47,48]. Consistent with this, our study found that patients with ACLF had a higher plasma levels of AST, ALT, TBil, and INR with respect to HCs and cirrhosis patients. H&E staining showed a larger area of hepatic parenchymal necrosis and more infiltration of inflammatory cells in ACLF patients than those in HCs or cirrhosis patients. According to qRT-PCR, levels of the proinflammatory cytokines (IL-1β, TNF-α, and IL-6) were extremely increased in ACLF patients compared with those in HCs and cirrhosis patients, which suggested a hyperinflammatory state in the liver of ACLF patients. Therefore, exacerbated production of these cytokines as a consequence of an over-activated immune system accompanied by systemic inflammation (“cytokine storm”) was observed commonly in ACLF. However, aiming to cure inflammation only may not be sufficient to reduce the mortality associated with ACLF because the widespread tissue damage goes beyond residual liver regeneration to maintain physical needs. Thus, some interventions to prevent cirrhosis from developing into ACLF could be a more effective strategy for reducing the mortality related to ACLF.

The innate immune system has a major contribution to ACLF development. The role of immune cells in the pathogenesis of ACLF has received considerable attention for many years. We undertook scRNA-seq on hepatic NPCs to ascertain the distribution of immune cells among HCs, cirrhosis patients, and ACLF patients. ScRNA-seq permitted more reliable and precise identification of subpopulations of immune cells according to the distribution of classical markers. The liver of ACLF patients had an increased proportion of monocyte/macrophages, but a reduced proportion of natural killer (NK) cells and neutrophils. Previous research reported that HBV-related ACLF patients have a reduced number and attenuated function of NK cells because of hepatitis virus toxicity and decreased production of interferon-γ [49], which are consistent with our results. However, Weiss et al. reported that ACLF was characterized by neutrophilia in peripheral blood [50], and the variation trend of immune cell subsets showed some difference with our study’s results, which may attributed to regional distribution. Our study detected the ratio of immune cell subsets in the liver while Weiss’s study focused on the ratio of immune cell subsets in the peripheral blood. Meanwhile, we also hypothesized an altered ratio of immune cell subsets in different stages of ACLF. In addition, activated macrophages or resident macrophages (e.g., KCs) secrete pro-inflammatory cytokines to amplify the pro-inflammatory signal, increase the recruitment of infiltrating immune cells, and further contribute to enhancing the inflammatory process [15,16]. Therefore, the mortality of ACLF is proportional to the activation and numbers of intrahepatic monocyte/macrophages.

Lymphangiogenesis has been shown to occur in chronic liver fibrosis in response to chronic inflammation [51,52]. LVs can help infiltrating immune cells to drain from the site of inflammation and accelerate inflammation resolution [11,12]. Hence, we explored the state of intrahepatic LVs among HCs, cirrhosis patients, and ACLF patients. Compared with that in cirrhosis patients, the number of intrahepatic LVs was decreased significantly in ACLF patients. Therefore, intrahepatic LVs may act as the “key point” when cirrhosis develops into ACLF. We supposed that having fewer LVs inhibited the drainage of intrahepatic infiltrating monocyte/macrophages, which led to a systemic hyperinflammatory state in ACLF patients. 

With the help of scRNA-seq, we also discovered a group of apoptotic and dysfunctional LyECs that were present almost exclusively in the livers of ACLF. As indicated in Figures S7 and S8, they tended to show high expression of genes related to the oxidative-phosphorylation pathway. This group of LyECs also displayed high expression of a series of genes related to apoptosis (e.g., S100A14, BAD, BAX, BID, PARP1, and so on) and was mainly involved in apoptotic processes, which may result in decreased LVs in ACLF patients. Meanwhile, we found that the expression level of PROX1 of LyECs in the ACLF group decreased to 61% of that in the cirrhosis group. A recent study reported that oxidized low-density lipoprotein impacted lymphatic permeability via reducing PROX1 expression in the NASH mouse model [38], which was not only consistent with our results, but provided evidence of LyEC dysfunction in our study. Then, the dysfunction of LyECs further resulted in impaired drainage of LVs, accumulation of infiltrating monocyte/macrophages, and more severe inflammation in ACLF livers. Ribera et al. reported that the upregulation of eNOS in the LyECs of cirrhotic rats caused impairment of lymphatic drainage, and another two recent reviews pointed out that phenotypic changes of LyECs often occurred in diseased tissues [53,54]. However, our research represents the first time to explore the dysfunctional alteration of LyECs in ACLF, which provides clues for further mechanistic research on ACLF. 

Targeting apoptotic and dysfunctional LyECs could reduce the incidence and mortality of ACLF. Therefore, understanding the cause and risk factors of ACLF is of great importance. We redefined infiltrating monocyte/macrophages to seven subpopulations and analyzed the potential regulatory network between monocyte/macrophages and LyECs through ligand/receptor analyses. The result showed that the group of LyEC2 (E4 cluster) was the core of the interaction network between monocyte/macrophages and LyECs. SPP1 secreted by monocyte/macrophages induced LyECs to undergo apoptosis and dysfunction via the SPP1/ITGB1 pathway. SPP1, also known as osteopontin, is involved in a variety of biological processes, including bone remodeling, innate immunity, acute and chronic inflammation, and cancer, and is closely associated with cell apoptosis [55]. Several studies have reported that SPP1 is a predictor and biomarker of prognosis of HBV-related ACLF [56,57], because the serum level of SPP1 is significantly higher in HBV-related ACLF patients compared with that in cirrhosis patients and HCs and is positively correlated with the ACLF severity, which is consistent with our results for SPP1 expression measured by qRT-PCR and IHC. Integrins, including ITGB1, are a family of heterodimeric glycoproteins expressed on tumor cells and the surface of activated endothelial cells. They mediate diverse biological events involving cell adhesion and signal transduction [58]. A recent study reported that ITGB1 mediated macrophage adhesion and promoted liver inflammation in murine nonalcoholic steatohepatitis [59]. Meanwhile, SPP1 can bind to integrins with high affinity, which activates signaling pathways to regulate cell proliferation, adhesion, invasion, and fibrosis [60,61]. Such evidence further supports our hypothesis that the SPP1/ITGB1 pathway has a major role in LyEC dysfunction in ACLF. We also tested the function of SPP1 for LyECs in vitro. We found that SPP1 increased the proportion of dead LyECs significantly, and that SPP1 treatment damaged the tube formation ability of LyECs in a dose- and time-dependent manner. 

**Figure 6:**
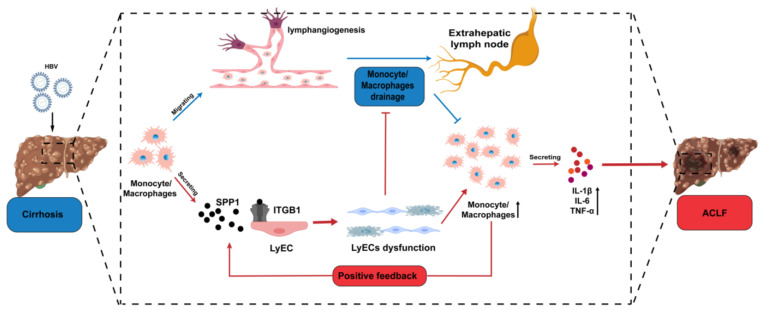
The diagram of the present study.

Taken together, our data suggested that during the development of cirrhosis into ACLF, some precipitating events (e.g., HBV infection) increased inflammation in the liver. A heavy hyperinflammatory state in the liver of ACLF patients caused inflammatory apoptosis and dysfunction of LyECs, which might be mediated by SPP1 secreted by infiltrating monocyte/macrophages. The apoptosis of LyECs resulted in decreased LVs in ACLF, which inhibited the drainage of intrahepatic infiltrating inflammatory cells, such as monocyte/macrophages. Meanwhile, dysfunction of LyECs further impaired the drainage function of LVs for inflammatory cells, and led to accumulation of intrahepatic inflammatory cells, which formed a positive feedback mechanism to promote a systemic hyperinflammatory state (Figure 6). Our results represent an important step towards understanding the pathophysiological mechanism of ACLF. 

However, our study also has several limitations. Our findings might be limited owing to the relatively small sample size. In addition, these results only took into account patients with HBV-related cirrhosis and ACLF. Therefore, research with more samples and different kinds of cirrhosis and ACLF are warranted in the future.

## 5. Conclusions

In conclusion, our study illustrated intrahepatic LVs and state of LyECs in ACLF patients and linked them to infiltrating monocyte/macrophages and systemic hyperinflammation. Our data deepen knowledge of the pathophysiological mechanism of ACLF at a cell-specific level and help to advance clinical and basic research on ACLF.

## Data Availability

The datasets generated during and/or analyzed during the current study are available from the corresponding author on reasonable request.

## Supplementary Materials

The following supporting information can be downloaded at: https://www.mdpi.com/article/10.3390/jcm11102910/s1. Figure S1: The cell viability for each liver; Figure S2: The quality control for scRNA-seq; Figure S3: KEGG analyses for high expression genes (*p* < 10^−50^) of E7 clusters showed a series of metabolic related pathways; Figure S4: KEGG analyses for high expression genes (*p* < 10^−10^) of LyEC1 clusters; Figure S5: KEGG analyses for high expression genes (*p* < 10^−10^) of LyEC2 clusters; Figure S6: The classical marker for each endothelial cells and epithelial cells cluster. CLEC4M expressed in liver sinusoidal endothelial cells (LsECs), PECAM1 expressed in vascular endothelial cells (VECs), EPCAM expressed in both lymphatic vessels cells (LyECs), epithelial cells and hepatocytes, CD34 expressed in vascular endothelial cells (VECs); Figure S7: KEGG analyses for high expression genes (*p* < 10^−50^) of LyEC2 clusters showed an oxidative phosphorylation pathway; Figure S8: The expression of genes related to oxidative phosphorylation pathway in ACLF and cirrhosis group; Figure S9: The tSNE and violin plotting of SPP1 in monocyte/macrophages; Figure S10: Increased mRNA level of SPP1 was found in cirrhosis and ACLF liver compared with HC (** *p* < 0.01) Table S1: The primer list in the study; Table S2: The clinical data of cirrhosis patients and ACLF patients enrolled in the study.
