# Supplementary material for: Single-Cell RNA Transcriptomics Reveals the State of Hepatic Lymphatic Endothelial Cells in Hepatitis B Virus-Related Acute-on-Chronic Liver Failure
Source: J Clin Med. 2022 May 20;11(10):2910. doi: 10.3390/jcm11102910 (PMC9143330; doi:10.3390/jcm11102910)
Supplement: Supplementary file 1 [file jcm-11-02910-s001.zip › jcm-1664730-supplementary.pdf]

Supplementary Figures

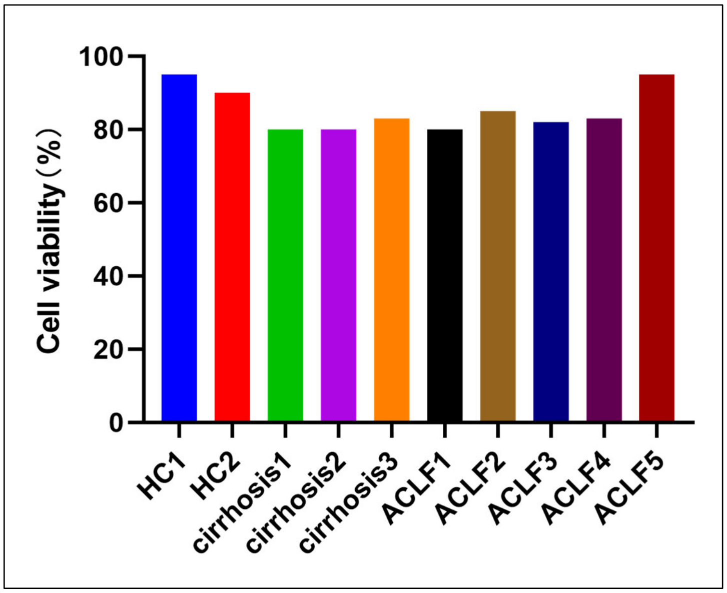

Figure S1 The cell viability for each liver

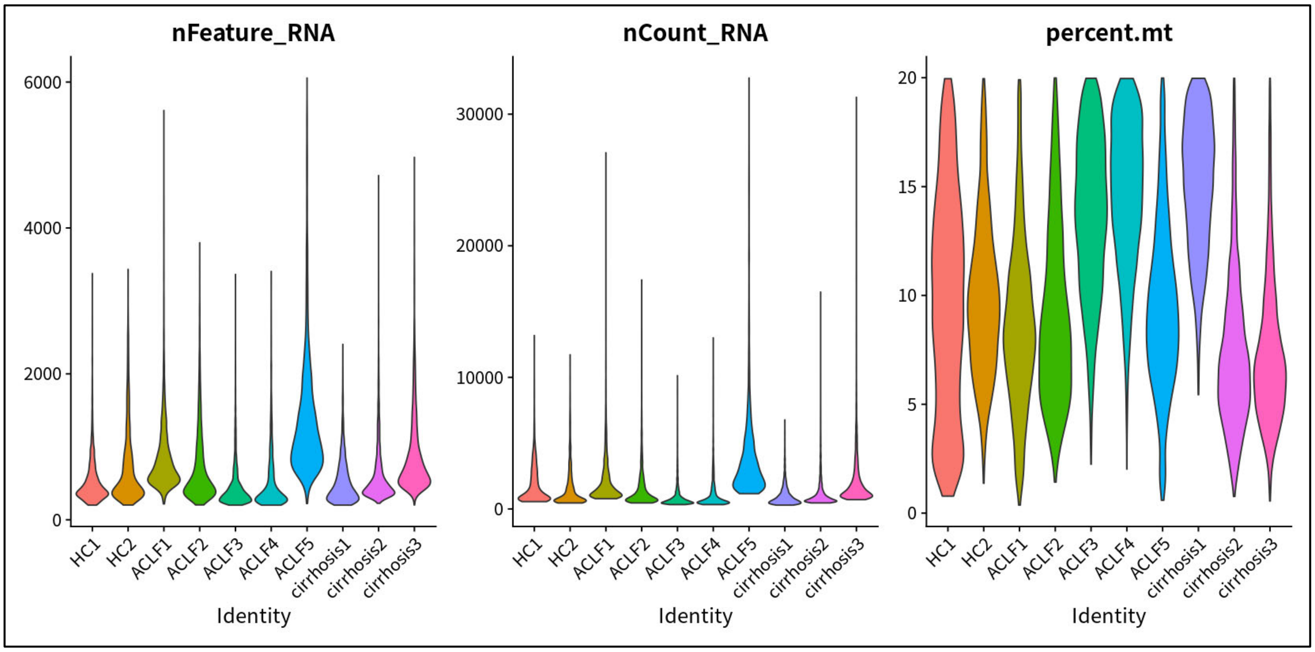

Figure S2 The quality control for scRNA-seq

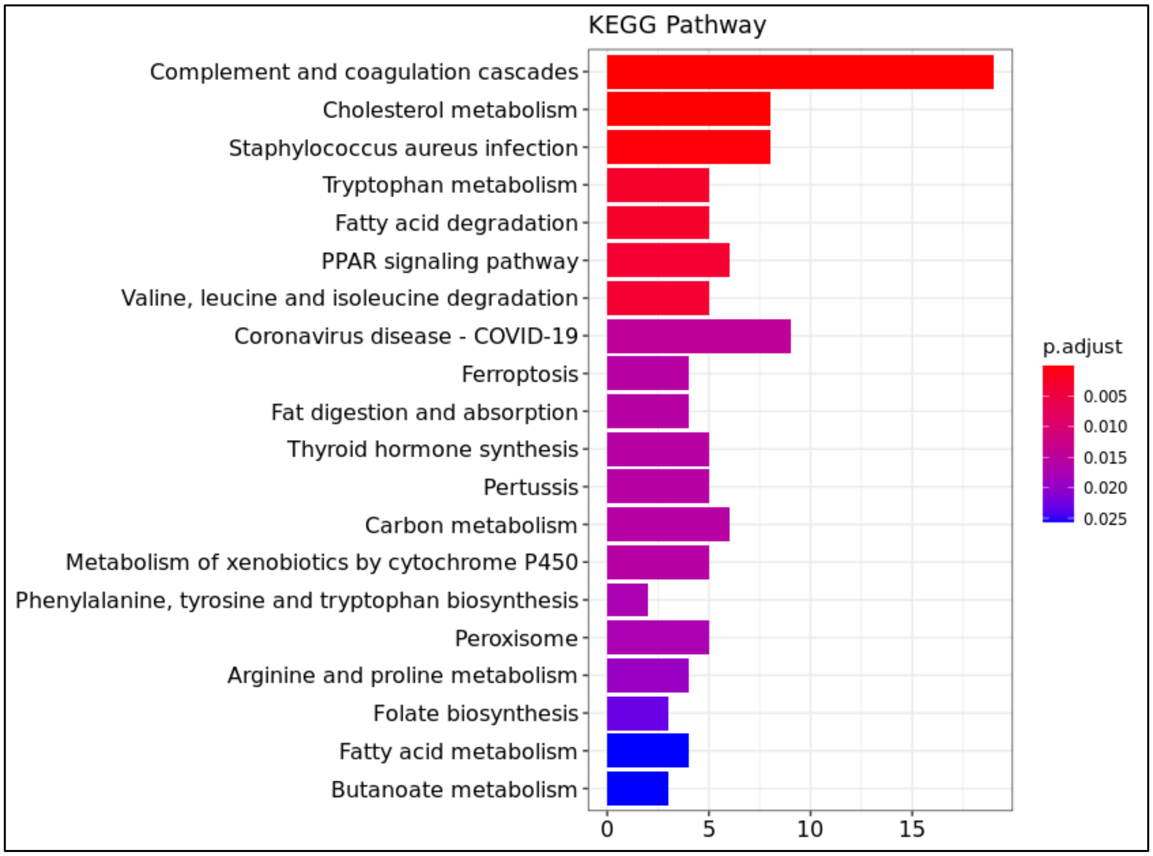

Figure S3 KEGG analyses for high expression genes ($p < 10^{-50}$) of E7 clusters showed a series of metabolic related pathways

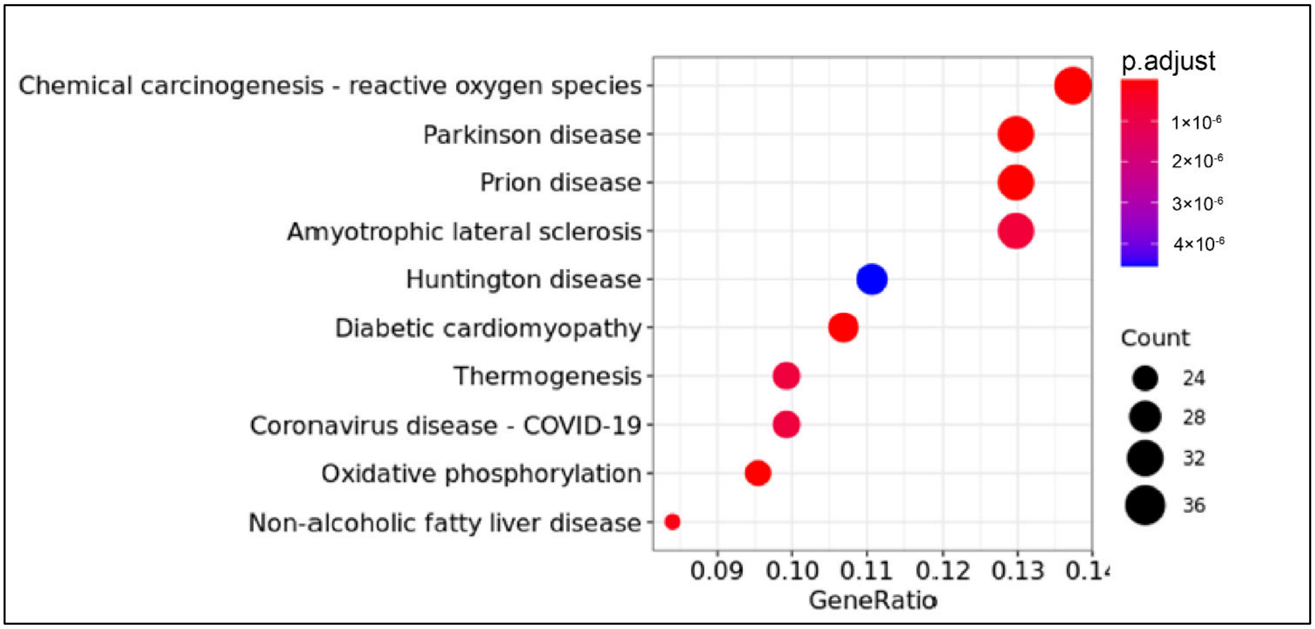

Figure S4 KEGG analyses for high expression genes ($p < 10^{-10}$) of LyEC1 clusters

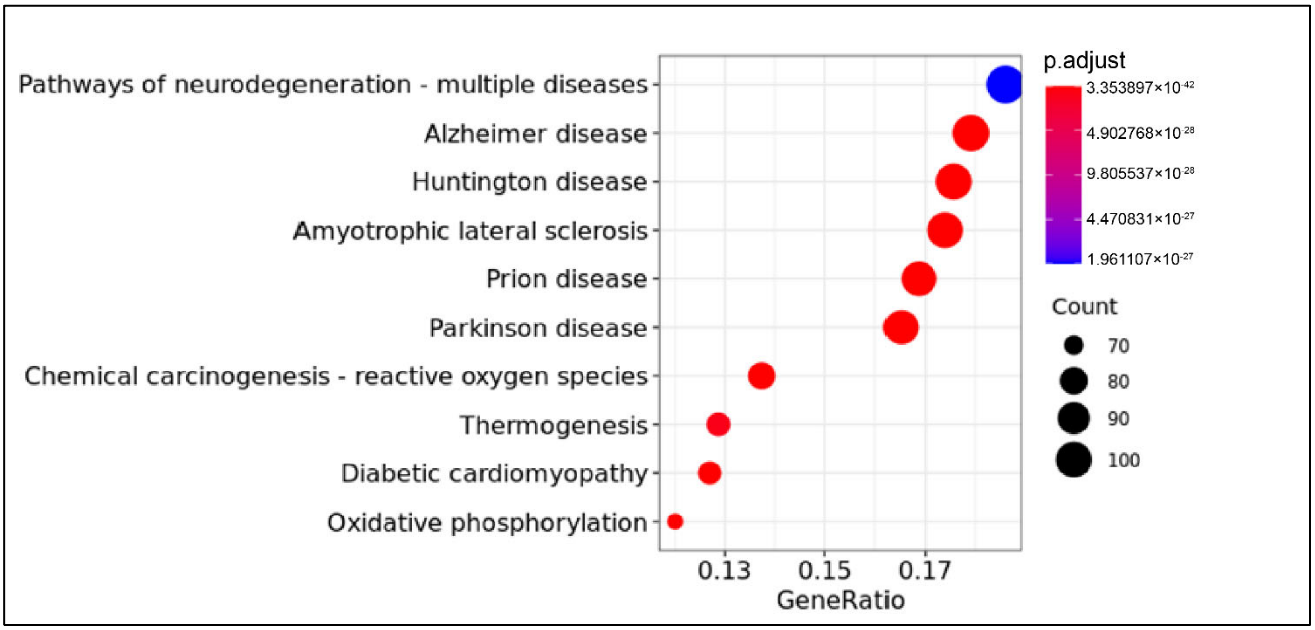

Figure S5 KEGG analyses for high expression genes ($p < 10^{-10}$) of LyEC2 clusters

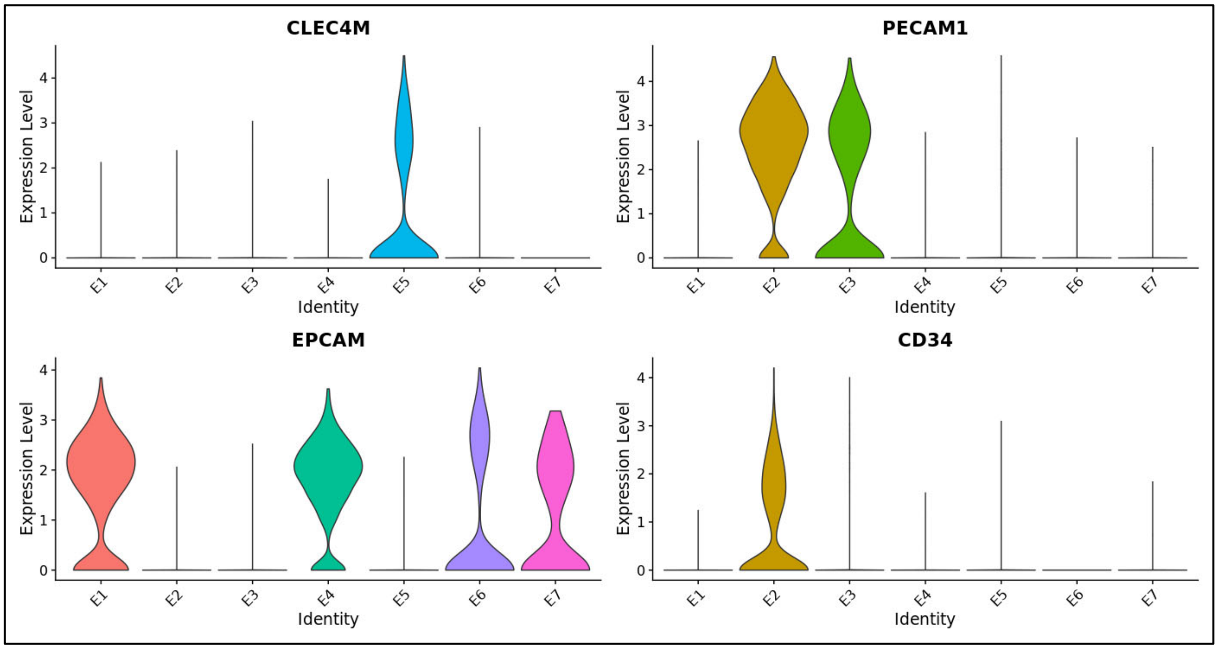

Figure S6 the classical marker for each endothelial cells and epithelial cells cluster. CLEC4M expressed in liver sinusoidal endothelial cells (LsECs), PECAM1 expressed in vascular endothelial cells (VECs), EPCAM expressed in both lymphatic vessels cells (LyECs), epithelial cells and hepatocytes, CD34 expressed in vascular endothelial cells (VECs).

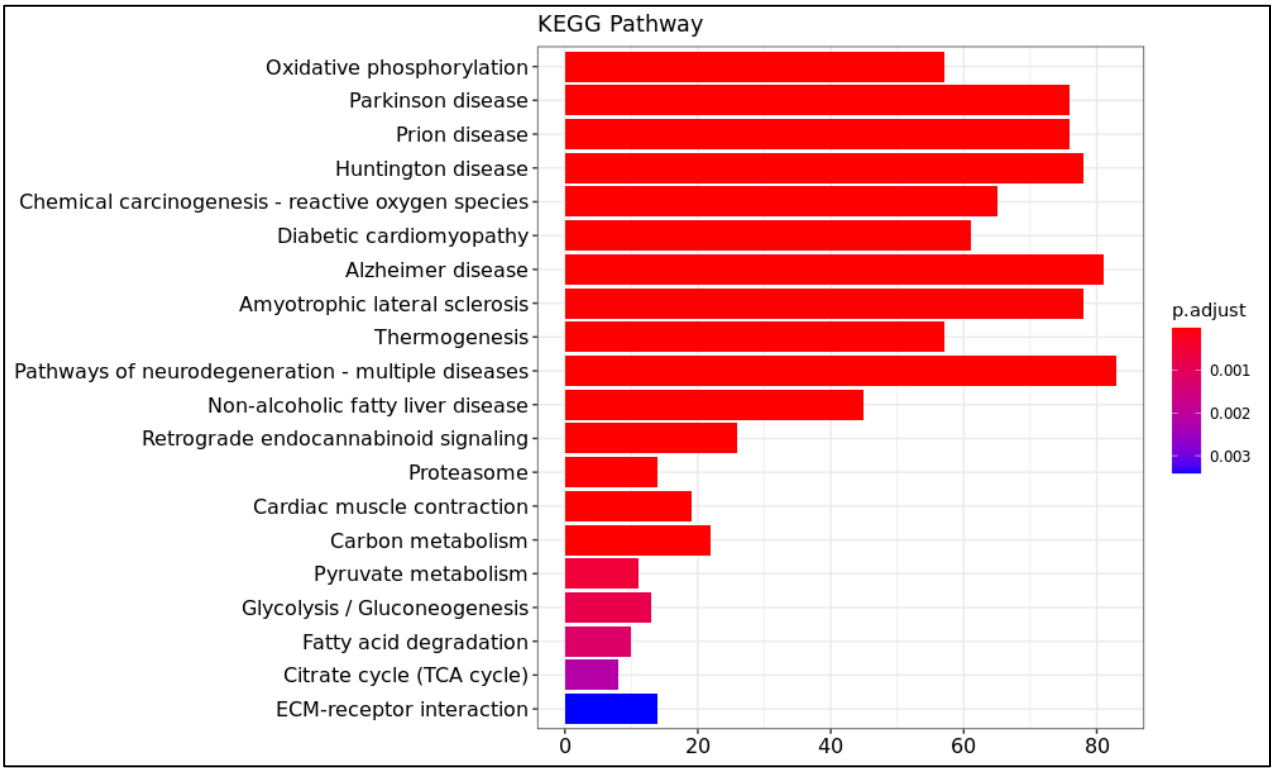

Figure S7 KEGG analyses for high expression genes ($p < 10^{-50}$) of LyEC2 clusters showed an oxidative phosphorylation pathway

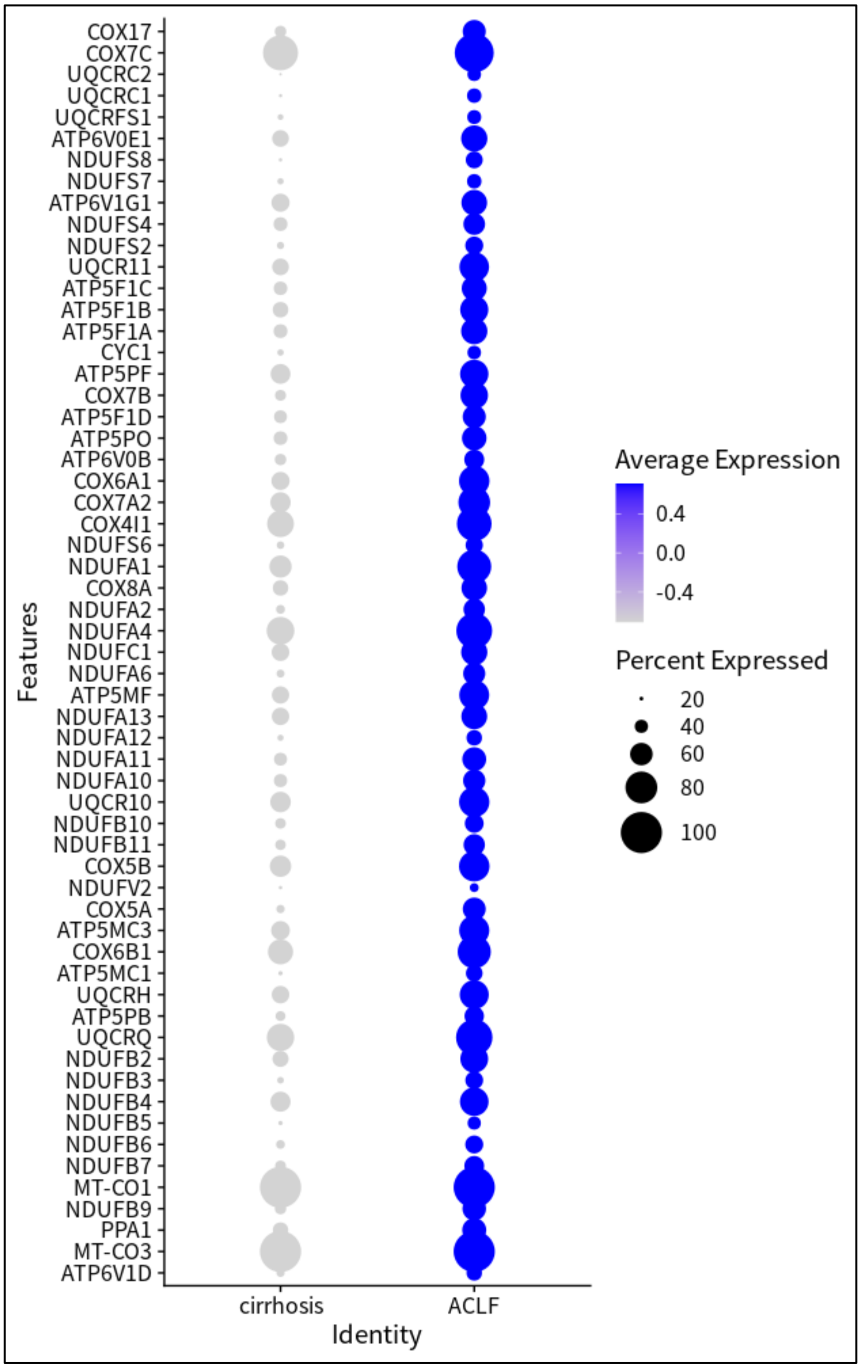

Figure S8 The expression of genes related to oxidative phosphorylation pathway in ACLF and cirrhosis group

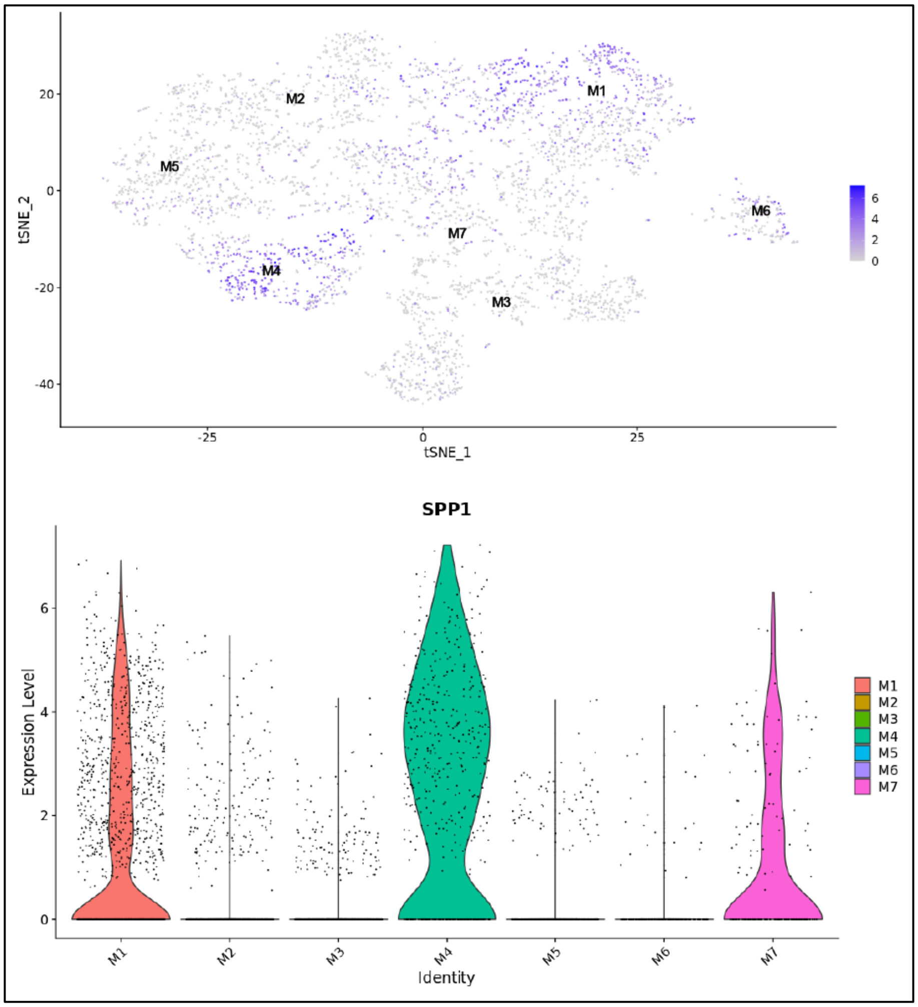

Figure S9 The tSNE and violin plotting of SPP1 in monocyte/macrophages

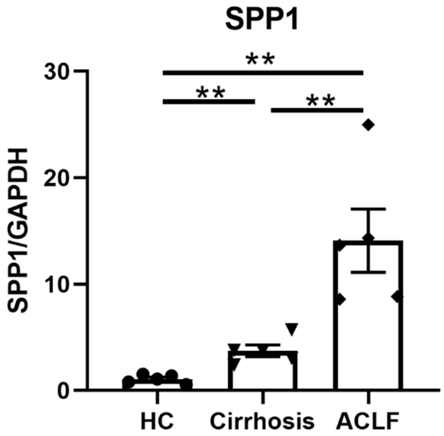

Figure S10 Increased mRNA level of SPP1 was found in cirrhosis and ACLF liver compared with HC ($p < 0{\cdot}01$**)**
